# HSAS-MD Analyzer: A Hybrid Security Analysis System Using Model-Checking Technique and Deep Learning for Malware Detection in IoT Apps

**DOI:** 10.3390/s22031079

**Published:** 2022-01-29

**Authors:** Alyaa A. Hamza, Islam Tharwat Abdel Halim, Mohamed A. Sobh, Ayman M. Bahaa-Eldin

**Affiliations:** 1 Computer & Systems Engineering Department, School of Engineering & Technology, Badr University in Cairo, Entertainment Area, Badr City 11829, Egypt; 2 Computer & Systems Engineering Department, Faculty of Engineering, Ain Shams University, 1 Elsarayat St., Cairo 11517, Egypt; mohamed.sobh@eng.asu.edu.eg; 3 School of Information Technology and Computer Science (ITCS), Nile University, 26th of July Corridor, Sheikh Zayed City 12677, Egypt; ihalim@nu.edu.eg; 4 Center for Informatics Science, Nile University, 26th of July Corridor, Sheikh Zayed City 12677, Egypt; 5 Faculty of Computer Science, Misr International University, KM 28 Cairo—Ismailia Road Ahmed Orabi District, Cairo 11828, Egypt; ayman.bahaa@eng.asu.edu.eg

**Keywords:** data security, triggers/actions, smart homes, software verification

## Abstract

Established Internet of Things (IoT) platforms suffer from their inability to determine whether an IoT app is secure or not. A security analysis system (SAS) is a protective shield against any attack that breaks down data privacy and security. Its main task focuses on detecting malware and verifying app behavior. There are many SASs implemented in various IoT applications. Most of them build on utilizing static or dynamic analysis separately. However, the hybrid analysis is the best for obtaining accurate results. The SAS provides an effective outcome according to many criteria related to the analysis process, such as analysis type, characteristics, sensitivity, and analysis techniques. This paper proposes a new hybrid (static and dynamic) SAS based on the model-checking technique and deep learning, called an HSAS-MD analyzer, which focuses on the holistic analysis perspective of IoT apps. It aims to analyze the data of IoT apps by (1) converting the source code of the target applications to the format of a model checker that can deal with it; (2) detecting any abnormal behavior in the IoT application; (3) extracting the main static features from it to be tested and classified using a deep-learning CNN algorithm; (4) verifying app behavior by using the model-checking technique. HSAS-MD gives the best results in detecting malware from malicious smart Things applications compared to other SASs. The experimental results of HSAS-MD show that it provides 95%, 94%, 91%, and 93% for accuracy, precision, recall, and F-measure, respectively. It also gives the best results compared with other analyzers from various criteria.

## 1. Introduction

The Internet of Things (IoT) connects multiple smart objects that work together automatically with limited human activity. With expectations of spreading to 75 billion devices by the end of 2025, IoT is one of the fastest-growing areas in the internet world [1]. As a result, IoT security and privacy have become one of the hottest topics. Because of the growing number of IoT applications, such as in healthcare, industrial, agriculture, and smart home applications, etc., there is increasing vulnerability to attack and violations of privacy due to the different types of devices and sensors. There are several attacks such as (a) physical attacks, (b) software attacks, (c) network attacks, (d) encryption, or data attacks that may occur in IoT [2,3].

The IoT application consists of several IoT systems that connect physical processes with digital communication, and these depend on edge-based (sometimes also known as event-driven) application. Event-driven application is responsible for taking the data from sensors such as motion sensors, contact sensors, and smoke sensors, and command from actuators such as smart power outlets, door controls, and smart locks towards providing different forms of automation [4]. IoT systems also build their infrastructure with (i) devices from the bottom up (ii) networking protocols, and (iii) IoT programming platforms [5].

One problem of IoT is the unequal status of existing platforms. As a result, the software stack designed for the applications responsible for monitoring and controlling all objects. Most of them do not have good essential tools and services to analyze security and privacy. In particular, third-party apps installed by end users to automate IoT devices’ tasks may include data leakage and abuse of permissions. Untrusted third-party apps lead to serious errors and misconfigurations which affect the behavior of IoT applications [6,7].

Security analysis systems (SAS) play an essential role in providing security and privacy, as they can analyze the behavior of IoT applications by detecting any malware, violations, or vulnerabilities. SASs use different program analysis techniques and methods of analysis (static, dynamic, or hybrid). The use of an SAS does not present any problem in discovering traditional malware, but what is dangerous is the emergence of new types of these malicious codes. According to science and business studies, around one million malware files are generated daily, and cyberattacks would harm the world economy by around 6 trillion USD yearly by 2021 [8].

Some SASs use static analysis such as [9,10,11], which analyzes the program without executing; this method has drawbacks, as some data may be lost during the analysis phase. Other SASs use dynamic analysis such as [12,13,14]. This analytical method discovers the code skipped by the static analytical methods, either because it is not created until rendering or cannot reach statically analyzable control flow [15]. Therefore, the hybrid analysis provides the best result to detect any malware, by avoiding static or dynamic usage drawbacks.

Static analysis extracts static features from source codes, including strings, opcode, N-grams, bytes, etc. On the other hand, dynamic analysis extracts dynamic features, including API traces, instructions traces, network traffic, etc. Therefore, the usage of hybrid analysis is the best tool for analysis to cover all features.

The main contributions of this paper are to introduce:

- HSAS-MD, a new SAS among the effective SASs, which is continuously being developed as a security compiler tool to act as a protective shield to detect malware. It depends on the PA of the IoT APP, which may automatically verify app behavior.
- HSAS-MD, a hybrid analysis which analyzes IoT Apps by extracting static and dynamic features based on model-checking techniques. It also utilizes deep learning to discover new malware and classify it to provide security, safety, and privacy by detecting any abnormal behavior.
- The evaluation of the HSAS-MD performance, which is measured by applying the proposed analyzer after enhancing CNN. The results of the proposed HSAS-MD were compared with similar SASs. HSAS-MD gave the best accuracy, precision, recall, and F-measure results.

Most of this paper’s sections are as follows: Section 2 presents an overview of the background and some basic concepts related to IoT security analysis. Section 3 illustrates the recent related works of the security analysis system for IoT and the research goals. Section 4 presents a proposed hybrid security analysis system (HSAS-MD Analyzer) with more details. The implementation and evaluation of HASA-MD are present in Section 5. Finally, Section 6 presents conclusions, limitations, and future research trends in the security analysis system for IoT. The relevant abbreviations used in this paper are shown in Abbreviations.

## 2. Background and Basic Concepts

This section presents an overview of the security analysis system (SAS) for IoT (Section 2.1), which includes some information about the Model-Checking Technique (MCT), Path Sensitivity, and Control Flow Graph (CFG). The basic concepts of the smart things platform are then presented in (Section 2.2), while (Section 2.3) shows the role of deep learning (DL) for IoT security.

### 2.1. Security Analysis Systems (SAS) for IoT

Most research has proven that IoT architecture is composed of three layers, which are: (i) the precision layer (or physical layer), which contains actuators, sensors, and intelligent objects; (ii) the network layer, responsible for linking, transmitting, and processing intelligent devices and objects; (iii) the application layer, which handles all applications that users interact with, such as healthcare and smart home applications. In addition, there are more famous control platforms which developers can use to construct smart apps that interact with sensors and actuators wirelessly, such as Samsung’s SmartThings platform [16], Apple’s HomeKit platform [17], and Amazon’s Alexa platform [18].

Most IoT security research tends toward the security of platforms, communication protocols, smart applications, and IoT devices. On the other hand, there are few studies on security from a comprehensive IoT perspective, including the interaction between smart objects and their applications and platforms [19]. IoT platforms acquire sensor and device data, then transform it into a lightway that can be easily consumed by other application software. This data must be processed and analyzed to make sure it is not malware. Event-driven technology is one of the technologies responsible for handling the communication between application, platform, and services by events.

Event-driven technology can specify the criteria to be fulfilled; to collect, evaluate, and continue to compare a significant amount of heterogeneous data in order to identify different situations within a given domain [20]. It is also an interactive system of objects managed by an event dispatcher (which acts as an intermediary between objects) through a message-passing mechanism. There are gaps in the event-driven technology; for example, (1) there are no access control mechanisms for object aggregation, event transmission, and event capture; (2) the effect of disfigured events is usually hard to control, and these gaps could easily expose you to various types of malware.

**Figure 1:**
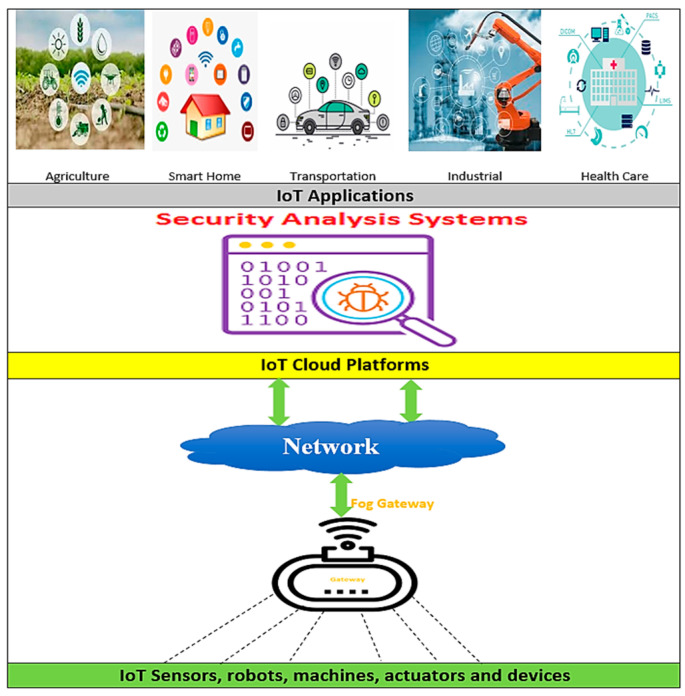
Security analysis systems for IoT applications.

Security analysis systems (SAS) focus on malware detection while transmitting or receiving data to/from IoT devices. IoT apps verify this behavior, as shown in Figure 1. The heterogeneity of IoT applications and platforms makes the malware publisher more accessible. Therefore, SAS has an efficient role in providing security for IoT applications.

An SAS depends on four factors to build an efficient detector: (i) the PA type used (static, dynamic, or hybrid); (ii) the PA technique used in the SAS, such as model-checking, program-slicing, taint-tracking, etc.; (iii) PA sensitivity, describing splitting data into uncertainties in the output such as context, path, API, etc.; (iv) characteristics of analysis, such as a control flow graph (CFG).

#### 2.1.1. Model-Checking Technique (MCT)

It is a significant challenge for IoT systems to prevent any infiltration by various malware types. The model-checking technique (MCT) is used in static and dynamic analysis to detect malware. This technique detecting malware belonging to the same family well and is also successful against malware based on obfuscation and polymorphic techniques. MCT identifies the sequencing of states that occur during the program’s execution and determines whether or not the sequences of states meet the safety property [21]. It scans and checks the behavioral property inside the source code to find any mistakes. The code is considered error-free if no errors are detected regarding a given property.

In [22], MCT is used for formal verification in IoT apps, including healthcare, communication protocols, security, and monitoring applications. MCT examines a system’s validity against properties and malware. Malware behaviors are manually extracted in this detection method, and behavior groups are structured using linear temporal logic (LTL) to represent a specific feature [23]. This enables flexibility in verifying all desired properties, and offers concrete counterexamples with linear temporal logic (LTL), which is helpful in studying why and how bad states exist. It can catch the holistic essence of the test’s multi-application connections and more effective—but not rigorous—checks [4]. MCT is distinguished from other techniques in improving system quality: (1) Testing is the most efficient and straightforward method of detecting problems; (2) Abstract interpretation and other program analyses are similar to model checking, in that they use algorithms to prove program properties automatically; (3) Higher-order proving is a strong and proven method for verifying complex systems [24].

#### 2.1.2. Control Flow Graph (CFG)

CFG is one of the directed graphs in which the nodes represent simple blocks (a linear sequence of program instructions of one entry point and one exit point), and the edges represent control flow paths. CFG has benefits, including: (i) capturing a significant element of code complexity; (ii) having a simpler representation form than source code. (iii) being able to directly list different improvement actions [25]. Each graph is generated using path sensitivity. It is sufficiently reliable for verification. It can justify branch connections, which are typically required to check the number of error reports obtained during the test. In [26], there are two symbolic states at a melting point in the control flow. It has the same ownership status, producing one symbol, the power, by incorporating their implementation cases in the data flow analysis. Symbolic states are otherwise treated separately as in the study of sensitive paths.

### 2.2. SmartThings Platform

SmartThings is one of the most common open developer platforms for the IoT, with many applications among the platforms available today. Smart devices deal directly with the central hub. An IoT application called Smart Apps, or the third-party Apps, a small Groovy-based system, allows users to optimize their homes using the devices’ capabilities. This type of app is the riskiest in IoT as it is untrusted. It may contain misconfiguration—even malicious intent—and bugs that present a threat. The SmartThings platform has some advantages which make it the best choice from the other platforms: (i) it has a set of 521 apps called Smart Apps. Specific competing platforms such as HomeKit, Weave/Brillo, and AllJoyn have fewer than 50 applications each in formative stages; (ii) it has built-in support from major manufacturers for 132 different devices; (iii) it shares the key principles of security architecture [27,28,29].

**Figure 2:**
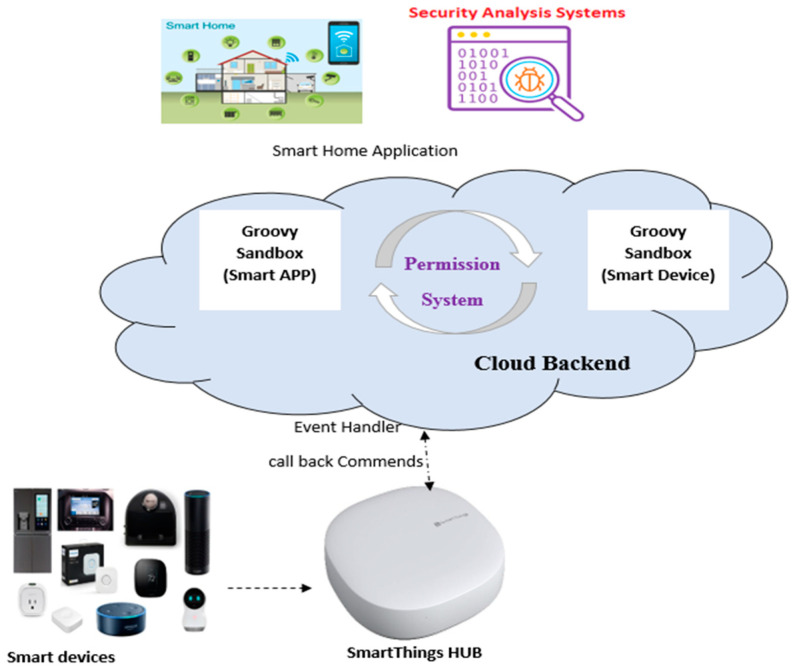
SmartThings Platform Architecture.

The SmartThings platform comprises three main mechanisms, as shown in Figure 2: (i) a permission system scheme that allows developers to define devices and user inputs needed for a specific device app at the time of launch; (ii) Smart Devices, a software proxy that runs the apps for hardware devices; (iii) SmartApps, a software proxy enabling users to manage their homes using device capabilities. Events are an integral part of the SmartThings platform. They allow SmartApps to respond to physical environment changes and create automated processes around them. SmartApps or Device Handlers do not explicitly create event instances. Instead, they are internally made by the SmartThings platform and passed on to SmartApps event handlers who have subscribed to those events.

### 2.3. Deep Learning (DL) for IoT Security

The SAS systems that use machine-learning or deep-learning methods are more productive than those that depend on program analysis techniques. They are significant in converting IoT systems’ security from simply supporting secure connectivity between devices to security-based intelligence systems. In [30], deep learning (DL) is a branch of machine learning that has advantages in previous studies at detecting security breaches. It has a high effect on malware detection due to learning more conceptual functions, reduced training complexity, increasing accuracy, managing massive datasets, and supporting feature extraction. In [31] a DL approach is proposed based on Stochastic Gradient Descent (SGD), which identifies attacks in the social IoT. The results show that DL methods outperformed more profoundly in every assessment aspect. A multitask deep-learning model for malware detection and classification was proposed in [32]. A combined feature set consisting of null-terminated tokens, API event plus parameter value, and API trigrams, were obtained from static and dynamic analysis. A deep feed-forward neural network was trained using a planned feature vector. In [33], CNN with a one-dimensional kernel (CNN-1D) is used to detect network intrusions. The detection of network intrusions aims to identify network traffic as either normal or an attack carrier. The information taken from network flow packets is used to classify the data [34]. 

The SAS’s main objective is to detect any malware present in the data sent or received between cloud backend platforms and smart mobile applications.

## 3. Related Works and Research Goals

This section presents an overview of various related SASs recently applied to IoT applications (Section 3.1). Section 3.2 gives the proposed analyzer’s research scope and objectives, and Section 3.3 presents the triggers/actions concept in the IoT app.

### 3.1. Related Work

IoT apps are convenient, but they expose us to new security risks. These risks may come from the collaborative behavior of several apps compared to traditional software applications. Early work used customized safety and security principles to detect these risks; however, these principles may not predict all device and application usages in a smart home, ending in false alarms. In [35], extraction sandboxes are used as a security measure in IoT. After analyzing a set of behaviors from various apps and devices, a sandbox is installed, reinforcing the idea that previously undiscovered behaviors are not permitted. As a result, the execution of malicious behavior introduced by software upgrades or concealed by strategies to resist program analysis is prevented. Therefore, there are two main critical issues in IoT applications. These issues are as follows: (i) verifying the codes by detecting any abnormal behavior in the IoT applications; (ii) detecting if there is malware or not. Therefore, the analyzers are built to solve these issues.

In addition, the analyzers have important metrics that play an essential role in improving their accuracy, such as analysis type and analysis technique. In [36], the author argues that static analysis continues to improve by using various methods to deal with the most advanced malware obfuscation techniques. However, there is a fundamental limit to what can be consistently detected to detect opaque constants; an obfuscation transformation has been created that obscures program control flow, hides access to local and global variables, and disrupts tracking values held in processor registers, using the proposed obfuscation method. Moreover, the opaque constant primitive can be implemented in a manner that may be difficult for any static-code analyzer to analyze. This therefore shows that static analysis alone may no longer be sufficient to identify malware. Instead, the hybrid analysis is the best for obtaining accurate results.

In [37], a framework was built for malware detection, focused on correlating and configuring static and dynamic API call sequences. Throughout their study, they examined the distinction and relationship between static and dynamic API call sequences by identifying various forms of malicious behaviors. After correlation and fusion, a hybrid vector space is developed for detection and classification to test their method’s efficacy. Finally, four classifiers were trained to detect/classify malware, including Random Forest, K-Nearest Neighbor, Decision Tree, and Extreme Gradient Boosting. In addition, [38] presented a framework that combines static and dynamic features into a single classification scheme. Function length frequency and printable string information are extracted and translated to vector representations for each executable file. First, all vector features are merged into a single vector for each executable. The resulting vector is then used as input to four simple classifiers.

Several surveys discussed malware detection using different techniques; for example, articles [39,40,41] offer a systematic and comprehensive description of machine-learning malware-detection strategies and DL techniques. It offers DL mechanisms categorized by the type of network input, according to the methods used to extract a feature vector representing the executable. The methods use the grayscale description of the executable as input, as well as use it to fed with the series of API function infection. these methods depend on describing programs according to a series of instructions, a byte stream, and data traffic.

In [5,41], security and privacy issues are presented in IoT platforms such as Samsung’s SmartThings, Apple’s HomeKit, Open-HAB, Amazon AWS IoT, and Android Things, that motivate program analysis techniques such as model checking, taint analysis, code instrumentation, and symbolic execution).

The code of an IoT application can be transformed into a sensor-device state-actuator platform structure with three types of common building blocks: permissions, events, and call graphs. It focuses on program analysis goals, i.e., sensitive data leaks and permission misuse. It also discusses the issues and challenges in IoT program analysis according to three points: firstly, according to IoT-specific issues such as physical channels, simulation and modeling of IoT programs, automated tests-case generation, multi-app analysis, interaction between IoT devices, and trigger-action platform services; secondly, according to IoT application idiosyncrasies such as RESTful APIs, and language-inherited operations; thirdly, according to analysis sensitivities, such as flow sensitivity, context sensitivity, field sensitivity, path sensitivity, and provenance tracking). It includes FlowFence, Saint, ContexIoT, SmartAuth, ProvThings, and Soteria. Many researchers are still conducting security studies for IoT applications to provide a practical overview of the security solutions for supporting security by applying many SASs, as shown in Table 1.

Analysis types (static, dynamic, or hybrid) and analysis techniques are essential for building security analyzers to detect malware [41]. Many security analysis systems (SASs) are based on static analysis. Soteria [10] is a static analyzer using the model-checking technique (MCT), that extracts a state model from the code of an IoT application to verify if an application or multi-app system respects security, safety, and functional properties. Another version is Soteria2 [42], a static analyzer using the convolutional neural network (CNN). It is a random walk-based traversal method for feature extraction that employs both density-based and level-based CFG labels to achieve consistent representation.

IotSan [4] employs the MCT and state model to check the approach to identify the causes of cyber vulnerabilities, and provides actual precedents to clarify such triggers. ForeSee [43] creates a multi-layer IoT hypothesis graph by simultaneously representing all of the critical components of IoT systems, such as the physical surroundings, devices, protocols, and apps. If there are any violations, the model checker can evaluate the created hypothesis graph to validate system security or generate attack paths.

There are other static analysis SASs that use MCT. However, they depend on extracting rule models, rather than state models, from the code of IoT applications such as IoTCOM [6], SIFT [44], iRuler [45], and TAPInspector [46]. They all utilize static analysis algorithms to automatically identify IoT app behavior from rule models.

The SASs based on dynamic analysis are IoTGuard [12] and IOTFUZZER [14]. IoTGuard works using the code instrumentation technique to analyze cod. It stores data of the apps in a dynamic model, then checks security issues on the dynamic model. However, IOTFUZZER identifies vulnerabilities to memory corruption in IoT devices without accessing their firmware images using dynamic analysis.

The use of static or dynamic analysis is not enough to detect all malware. Every type of analysis cannot cover all malware. PATCHEC-KO [47] is a hybrid analyzer that depends on static and dynamic analysis. It optimizes deep learning and hybrid binary analysis to execute multi-platform binary code similarity analysis to identify vulnerabilities without high-precision source code access. It is noted that hybrid analysis is more effective in detecting malware after studying different SASs. Moreover, MCT is more effective at detecting malware in IoT apps.

In this paper, the proposed HSAS-MD based on MCT uses hybrid analysis types (static and dynamic) and hybrid models (state and rule) to avoid the disadvantages of using one analysis type or analysis model. These hybrid analysis and hybrid models will increase the accuracy malware detection.

### 3.2. Research Scope and Objectives of the Proposed Analyzer

**Figure 3:**
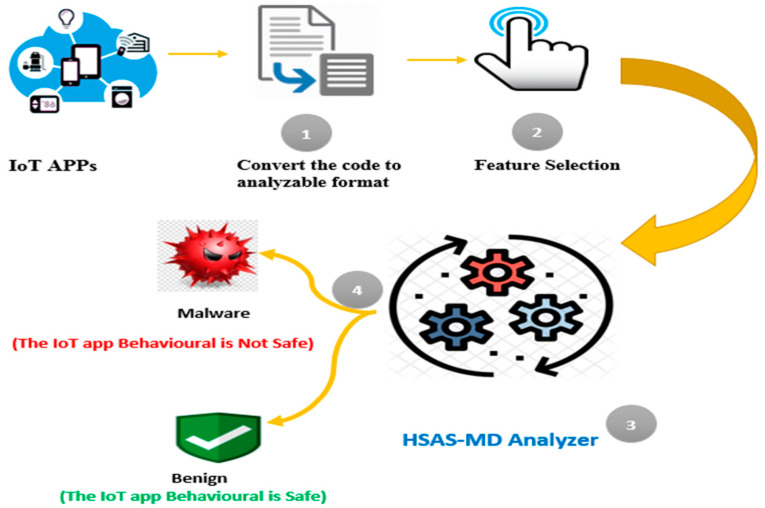
The research scope of the proposed analyzer.

The research scope depends on converting the source code of the third-party new IoT apps to analyzable formats, as shown in Figure 3, then selecting the static features’ “actions”. The proposed HSAS-MD analyzer works on these features using the CNN model to detect malware in these features, then verifies if there is any abnormal behavior using MCT.

The proposed analyzer’s main objectives are to detect vulnerabilities in supporting IoT apps’ security through detecting malware and classifying them, as well as, using CNN, to detect any abnormal behavior (conflict actions) in the IoT application using MCT, such as breaches due to connectivity or device failures, misconfiguration problems, data leakage, and physical security breaches.

### 3.3. The Trigger/Actions in the Smart Home Apps’ “Third-Party App”

IoT environments consist of various applications that deal with others through commands (trigger, conditions, and actions) to operate or control devices and sensors. Every IoT app has rules specified using “an event-condition-action paradigm.” These rules represent the behavior of IoT APPs for the proposed HSAS-MD as groups of triggers and their corresponding conditions and actions. The rule model represented is Rh = <Th, Ch, Ah>, explained as follows:

- **Triggers (Th):** Cyber or physical events that devices transmit to smart homes, such as the activation of a motion sensor which triggers the rules;
- **Conditions (Ch):** If a rule may apply, the logical predicates are evaluated on the current status of the devices. For example, a rule runs only if the system is in “home” mode;
- **Actions (Ah):** When the conditions are satisfied, the rule changes the state of one or more devices, leading to a physical change, such as the activation of a light switch.

Each rule attribute (Th, Ch, Ah) can be represented as a triple of a device D(α), an attribute A(α), and a set of values V(α), in which α is a number from 1 to n [6].

**Figure 4:**
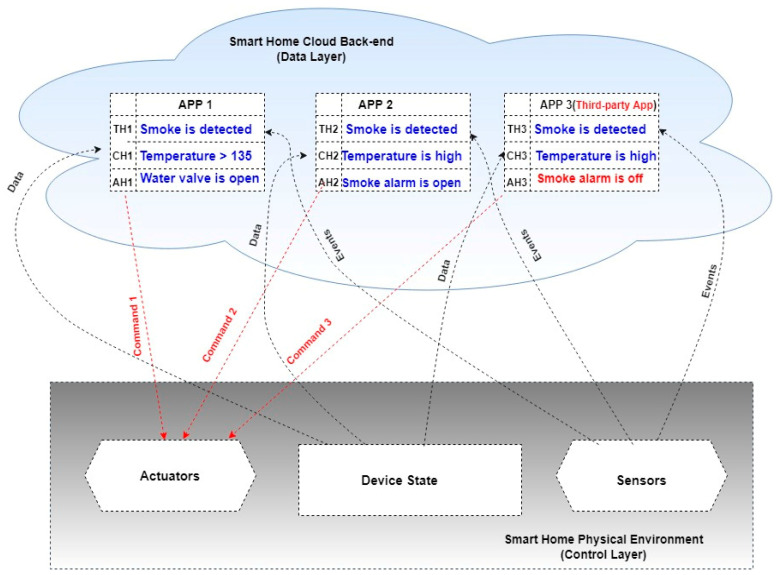
Smart home apps, including malicious third-Party apps.

The main problem is that end users can use untrusted apps, including malicious commands. This app is called a third-party app in an IoT environment. If this app contains malware (malicious commands), it will lead to unpredicted behavior of the other apps, devices, and sensors connected with it, as shown in Figure 4.

The smoke alarm application is one of the typical applications used in smart homes that end users may install. The main goal of this application is to detect smoke and act as quickly as possible. This application consists of a smoke-detection-alert system, a water valve (on the lower floor), and a light switch (in the living room). Operating this application issues a smoke alarm, and the lights open when smoke is detected. When the high-temperature level is reached, it opens the water valve to operate the fire sprinklers; finally, in the event of fire control, it turns off both the alarm and the water valve. Furthermore, the application is launched when the smoke detector battery is low. If malware breaches this application, it will make the application work. However, there is no reason to operate it, and nor does it use it in the event of a possible fire that causes great danger. Therefore, the presence of an SAS is more critical for the purpose of supporting security and protecting other IoT applications from receiving any actions from a malicious third-party app.

Events generated from the sensors are forwarded to the backend using software proxies, as shown in Figure 2; these events invoke triggers defined in the rules. If the present state of the cyber-physical system meets the conditions of a given rule, the rule initiates one or more actions, which are transmitted as commands to devices [5]. Therefore, it is important to detect the malware actions from the rules in the early stages to save time in the static analysis process.

Due to the various threats which are generated from the interaction of various apps, it is essential to consider the risks created by event-condition-action automation. Therefore, the security of the IoT platform requires an understanding of the interaction between two rules of APPS [45].

These two rules may include risks as bugs and malware. These risks are classified into various classes. In [6,46], the interaction between the two rules was organized into seven classes: T1 (Action–Trigger); T2 (Action–Condition when matched); T3 (Action–Condition when not matched); T4 (Self-coordination), T5 (Action–Action (Conflict)); T6: Action–Action (repeat); and T7 (Exclusive Event).

**This paper** focuses on the rule of the Action conflicts using a deep-learning CNN model. This rule means that two rules conflict with each other’s actions (ai =: −a_j_, in which ai is the action of Condition 1, and a_j_ is the action of Condition 2. They operate on the same device and attribute but have different values, such as turning on or turning off at the same switch). This risk can lead to the system entering an unstable or unknown state with the same latency of rules.

In [48], the authors present a conflict-classification survey of the interaction of IoT apps using model checks and formal notations due to the risk of conflicting rule issues. The Action–Action conflict rules can be represented as if there are two rules—R1 and R2—which are triggered by a similar event to access the same device D(α) and an attribute A(α), but with conflict values V(α), as shown in Box 1.

Box 1 **The interaction between two rules.**

**R_AC_ (R_i_, R_j_) ≡ (∃ A_h1_∈ A_Ri_; A_h__2_∈ A_Rj_; R_1_, R_2_∈ R_AC_. (match (A_h1_, A_h2_) ∧ (V(_α1_) ≠ V(_α 2_)) ∧ T _1_ ∗ (R_1_, R_i_) ∧ T _1_ ∗ (R_2_, R_j_) ∧ ((R_1_ = R_2_) ∨ sibling (R_1_, R_2_)))).**

Where:

R_AC…_ Rule of action conflicts

R_i…_ A series of rules, *i* = 1 to N

R_j…_ A series of rules, *i* = 1 to N

R1_…_ Rule _1_

R2_…_ Rule _2_

A_h__1…_ Action of rule 1

A_h__2…_ Action of rule 2

Vα _1…_ Value of the device in the rule 1

Vα _2…_ Value of the device in the rule 2

T_1 …_ Trigger 1

sibling (R_1_, R_2_) _…_ The two rules R_1,_R_2_ have the same trigger (T_1_) and a set of non-exclusive conditions for them.

This paper, HSAS-MD, focuses on using hybrid analysis to convert source code to an analyzable form. The CNN model extracted the malware by checking the actions in the rules before installing the app and verifying the app’s behavior using MCT.

## 4. The Proposed Hybrid Security Analysis System Based on the Model-Checking Technique and Deep Learning (HSAS-MD Analyzer)

This section presents the proposed hybrid security analysis system (HSAS) using model checking and deep learning (MD). An efficient SAS is one of the most critical issues related to IoT app security, serving as a security compiler. In a successful SAS, three factors must be considered: (i) Detecting malware (action conflict) at an early stage of analysis; (ii) Identifying and classifying new malware using the CNN model; (iii) Verifying the new IoT app’s behavior.

**Figure 5:**
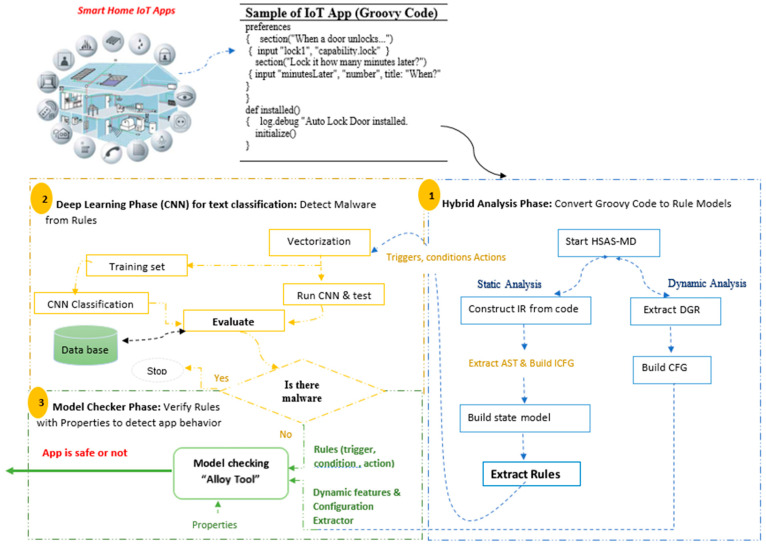
HSAS-MD analyzer phases.

The proposed HSAS-MD consists of three phases to detect malware early, as shown in Figure 5: hybrid analysis, the deep-learning CNN model, and the model checker. Each phase has an essential role in the proposed analyzer. The main goal of the hybrid analysis phase is to convert the source code (groovy code) of IoT apps to an analyzable format before using MCT. The output of the dynamic analysis is the dynamic features and configuration extractor which will be verified directly by the final phase using the model-checking technique. However, the output of the static analysis is the rule model, which consists of triggers, conditions, and actions. These will be formatted in a vector-based format. It will then be used in the following critical phase: the deep-learning phase.

**Figure 6:**
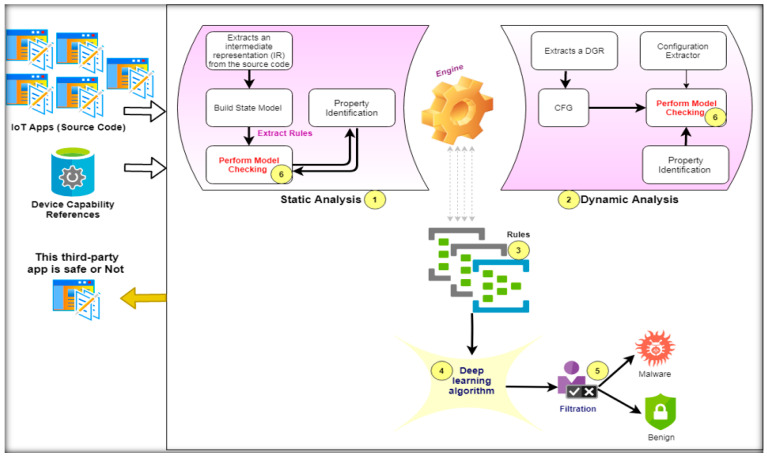
HSAS-MD Analyzer Architecture.

The main architecture of the proposed HSAS-MD is composed of six phases, as shown in Figure 6. (1) Static analysis phase is responsible for extracting rules from the source code of the IoT APP; (2) Dynamic analysis phase is responsible for extracting dynamic CFG from IoT app source code; (3) Rule model phase is responsible for verifying the rules; (4) Deep-learning (DL) phase is responsible for detecting and classifying features; (5) Filtration phase is responsible for deciding the final decision of whether the feature is malware or benign; (6) Finally, performing model-checking technique for verifying whether app behavior is safe or not in each analysis phase.

This second phase uses CNN for text classification. It depends on checking the actions from the rule model extracted from the first phase before sending them to IoT devices. The CNN for text classification detects malware early, and it stops app analysis if there is malware (malicious actions). This early-stage (CNN) saves time for the SAS to analyze another IoT app. If there is no malware, the actions from the rules will transmit to the model checker’s final phase.

The third phase is the model checker, which verifies the rules after also verifying dynamic features the configuration extractor with the properties of the apps from the CNN phase. The output of this phase is the decision of whether the behavior of the app is safe or not. All steps of the proposed analyzer phases can be illustrated as follows:

**Step 1**: Convert the app’s source code to analyzable form using phase 1 of the proposed analyzer.

- Static analysis: Start converting code to IR, extract AST, and build ICFG to make the state model, then convert the state model to the rule model form.
- Dynamic analysis: Extract DGR to build CFG.

**Step 2**: Start phase 2 with the extracted actions from the rule model.

**Step 3:** Convert the rules that contain actions to be vector-based.

**Step 4**: Run deep-learning “CNN for text classification” with the vector-based and test actions.

**Step 5**: Evaluate the output of the CNN. If there is malware (malicious actions), stop the analysis process; however, if there is no malware, phase 3 will operate with these static features.

**Step 6**: Start phase 3 using the model-checking technique (MCT) to verify the app’s behavior. It will check the static and dynamic features with the properties that describe all specifications of this app. Therefore, the proposed HSAS-MD analyzer is concerned with detecting security problems in IoT apps and detecting and classifying malware.

### 4.1. Static Analysis Phase in the Proposed HSAS-MD

**Figure 7:**
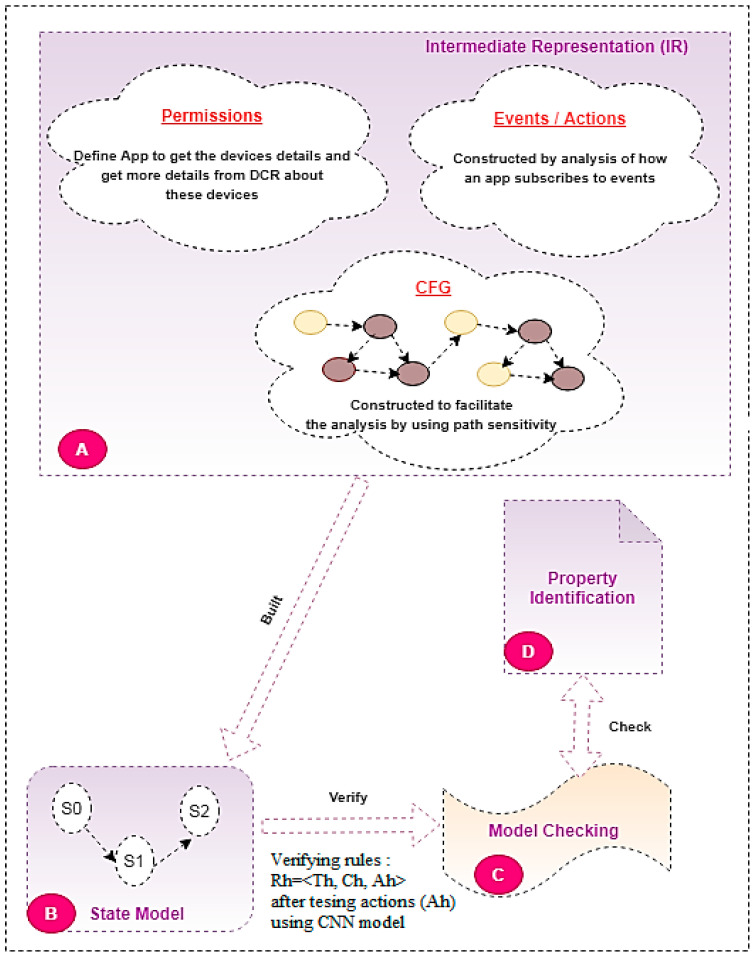
The static analysis phase in the HSAS-MD.

The static analysis phase depends on converting the IoT apps’ codes to a rule model that includes triggers, conditions, and actions before using the model checker, as shown in Figure 7. The static analysis phase depends on enhancing deep learning to check actions before using a model checker. It is composed of four steps: Step (A) is to extract an IR from the source code of an IoT application, including the event handler methods, entry points, and call graphs; Step (B) builds a state application model that includes its states and transitions from the IR, then converts the states to the rule model; in Step (C), model checking used to check that the app’s behavior is in line with those properties developed in step (D) when running independently, or interacting with other applications. Each step are described in more detail below:

Step (A) is the intermediate representation (IR). The IR has several advantages: it allows precise modeling of the application’s life cycle, removes parts of the code that are not relevant to property analysis, and finally enables the efficient extraction of states and state transitions from implementation. It consists of three mechanisms to convert the source code successfully to IR. The IR needs to access permissions, obtain events/actions, and build CFG. (i) Permissions are responsible for obtaining all information from DCR about the IoT application devices; (ii) Events/Actions represent the relation between events and actions (when an event occurs (is triggered), a related action is taken); (iii) CFC represents the relationship between entry points and their corresponding functions in the target application and facilitates analysis using path sensitivity. IR constructed via AST represents the Groovy source code of the IoT app. Analyzing the AST will build a call graph and API methods as a CFG for each local method using path-sensitive analysis [49]. Additionally, the AST visitors analyze the expressions from AST nodes that are built during the compilation process to create ICFG in order to extract the state model.

Step (B) is the state model representing the codes to a set of states from IR to detect violation from the interactions of state models between apps. Every state model will then be converted to a rule model: Rh = <Th, Ch, Ah>. The conversion from a state model to a rule model is used for the first time in an SAS. The rule model is better than the state model method at detecting violations via testing actions. The state model is formulated as (Q, S, δ), where Q is a series of states. Q represents the device’s attributes that change due to the event handlers; S represents a series of transition labels representing the events; δ is a function of the state transition using the Cartesian product of the attributes of its device. The state model (Q, S, δ) is created from the physical IoT device’s manufacturer’s specifications <device, attribute, value>. Each device has its attributes. Each device’s attributes (conditions in the rule model) have specific values (actions in the rule model). Therefore, the rule model Rh = <Th, Ch, Ah> is matched with <device, attribute, value>.

The advantage of the state model is the filtration of the deterministic and non-deterministic states using an SMT solver. This is essential to verify the path conditions [50]. The deterministic states represent the conditions for the safe operation of an IoT device. However, the non-deterministic states represent the safety violations. Therefore, it is an important step for using the state model. However, the state model is not effective with an increasing number of IoT devices, and malware actions were difficult to detect from the state model. In [10], two challenges to extracting states are listed: the first is state explodes, and the second is the precision of the state model is low as it depends on approximation. Therefore, the rule model is extracted from rewriting the logical equation: rl for rules, and crl for conditional rules [45].

For example, from app 1, as shown in Figure 4, when smoke is detected and the temperature is >135, the handler method is responsible for opening the water valve. From analyzing the handler method, there is a transition with the smoke detection and temperature >135 event labels from the “smoke undetected and valve closed” state to the “smoke detected and valve opened” state. The values (actions) in this app are “valve opened” and “valve closed”.

The property description needed in the static phase is in Step (D), the property identification. These properties are configured in temporal logic to be checked with the generated model of the target app using the model checker [51]. After discovering malware in actions (rule model), a group of security properties was generated to be validated using a model checker [46]. Some of these properties can be configured by the user. These properties were classified into three types: safety (S), liveness (L), and general (G), written using LTL or CTL. Examples for safety properties are as follows: S1, the water valve must be closed if a leak is detected; S2, the door must always be locked when the user is not home; S3, the alarm must always go off when there is smoke. Examples for liveness properties are as follows: L1, after 9 pm the light will eventually be turned off; L2, after 1 h the door will be locked. Examples for general properties are as follows: G1, do not turn on the living room light when no-one is home.

Finally, model checking (C) is responsible for verifying the code from the rule model with the corresponding properties, which is discussed in the next Section 6. More details for the workflow of the static phase are shown in Algorithm 1.


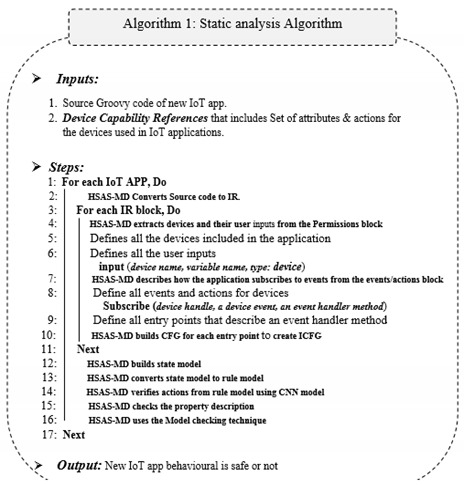


### 4.2. Dynamic Analysis Phase in the Proposed HSAS-MD

**Figure 8:**
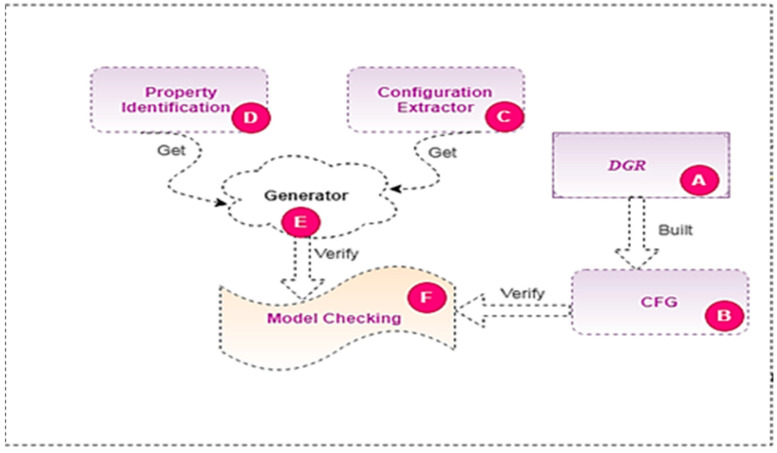
The dynamic analysis phase in the HSAS-MD.

The dynamic analysis phase focuses on dynamic features. It differs from the static phase, as dynamic analysis depends on understanding how the code performs during the execution process. This phase contains four steps, as shown in Figure 8.

More details for the workflow of the static phase are shown in Algorithm 2.


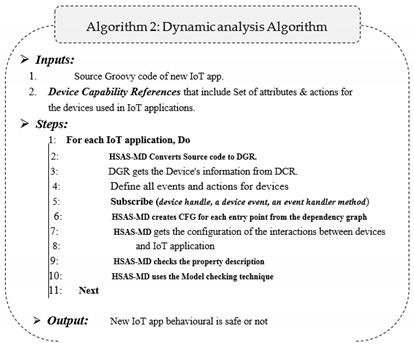


Step (A) is DGR. It collects all device attributes and actions from DCR and extracts the dynamic features. It then converts the dynamic features to a set of dependency graphs representing the interactions between event handlers of the app and reducing all unnecessary event handlers. Step (B) is the state model, an intermediate module that converts the output of dependency graphs to the representation of CFG. Step (C) is the configuration extractor, responsible for extracting all configurations related to the application and the connected devices. A set of property descriptions is stored in step (D), referred to as property identification. These properties are the same as in the static phase. In step (E), the generator is responsible for matching the configuration and its related properties, as in the static phase. Finally, Step (F), model checking, is responsible for verifying the code to ensure it does not contain any abnormal behavior.

### 4.3. Rule-Model Phase in the Proposed HSAS-MD

The third phase in the HSAS-MD is the rule-model phase. The smart home model includes a set of IoT apps. Each consists of a group of rules; these rules describe the function of the IoT app and the related devices if they communicate physically with each other. These devices include sensors, switches, thermostats, etc. However, if the IoT app communicates virtually through the cloud, the direct mapping used to trigger the virtual device or scheduling method is used by utilizing rule calls to invoke the second rule after a delay run the API from SmartThings platform apps.

The HSAS-MD extracts the rule model using the model builder from the static phase. It generates a formal rule-model representation, as shown in Table 2, including six slots which describe each rule. The trigger action rules play an essential role in discovering the bugs through the interactions between the rules.

Each rule includes three main elements: the trigger, condition, and action. All of them are described as follows: capabilities, attributes, and value, as shown in Table 2. The trigger starts when an event happens. The event represents the temperature, motion, state change, etc. The value of the trigger is a Boolean constraint, e.g., active, true. Some rules do not include a value in the trigger, such as AutolockafterXminutes. As shown in Table 3, the condition is essential in the rule.

The condition is represented as a Boolean such as true, value on. The condition is a Boolean constraint representing the states. When all states in the conditions are satisfied, the rule can be executed. Regarding actions, there are three types. The first type of action is one where the attributes do not change, such as sending a mobile notification. The second type of action depends on time. The third type of action is affected by actuators. This type changes the value of the attribute, such as turning on or locking something.

The HSAS-MD focuses on the third type of action; the value of attributes may include a conflicting value. This value may be changed when software is updated or by the end user to give a contradictory value of the desired actuators. These action-conflicts lead to serious situations in IoT apps.

To further illustrate the action conflicts, the AutolockafterXminutes app includes two rules (R0 and R1).

The first rule (R0), assigned to lock1.capabilities and attributes are found with no value.

The condition value in this app is to cap_lock_attr_lock_val_locked. If the condition of lock1 is already completed, the action value of the state will be cap_runIn_attr_runIn_val_on.

The second rule (R1) is assigned to the state, starting with the condition of the cap_runIn_attr_runIn_val_on. This condition depends on the same action of R0. The action will then be cap_lock_attr_lock_val_lock. The value of the action plays an essential role in making the app do its function or make unwanted behavioral. This phase therefore has a vital role in representing the rules Rh = <Th, Ch, Ah> as features using vector-based.

As shown in Table 3, this table is a dataset of rule-based IoT apps that includes ten applications. Each application includes its capabilities and rules. The capabilities contain the sensors and actuators.

Each application may include one rule or more rules depending on the task of the application. Each rule consists of a vector of triggers, conditions, and actions. The trigger has three elements: the capability object’s name, attribute, and value. Additionally, conditions and actions have the same elements.

The HSAS-MD focuses on the value of actions. However, it uses the word2vec model before using CNN to convert the Th, Ch, and Ah from words to vectors. The rule model, including its elements (capabilities, attributes, and values), will then be fed as an input to the DL phase (CNN) as features. From the training dataset of the IoT apps, when the condition elements (capabilities, attributes, and values) are matched, the value of actions can be detected using CNN. The early detection of whether the app has malware or not saves time of analysis.

### 4.4. Deep-Learning (Dl) Phase in the Proposed HSAS-MD

The proposed HSAS-MD analyzer uses the Convolutional Neural Network (CNN). CNN is one of the DL algorithms that can typically classify text [52,53,54,55] by taking a feature vector, assigning significance (trainable weights and biases) to various aspects in the input feature, and distinguishing one from the other. The preprocessing time needed for CNN is lower than for other different classifiers. CNN is capable of understanding these filters/characteristics.

**Figure 9:**
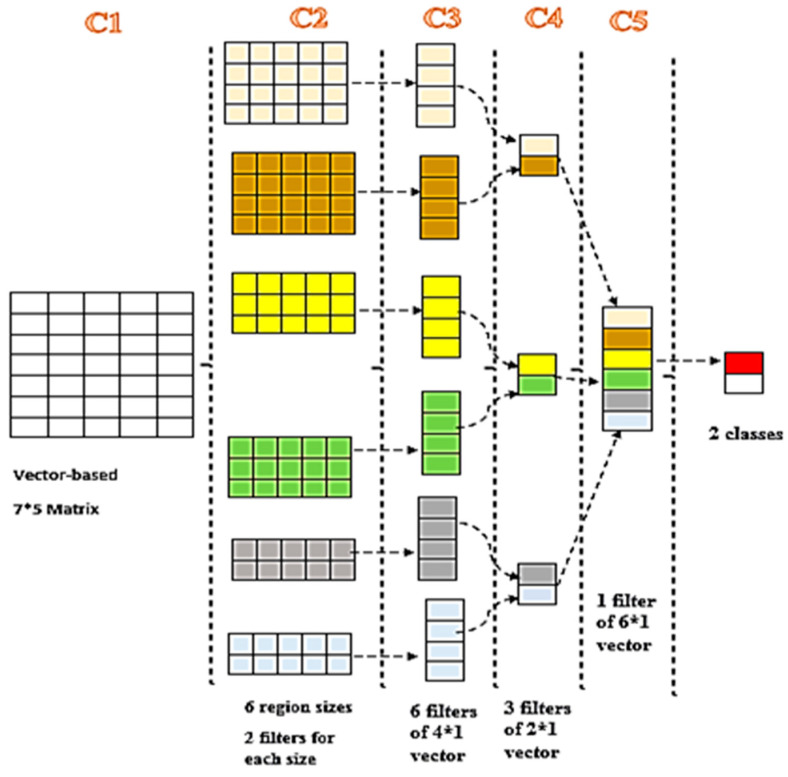
The DL phase in the HSAS-MD.

Features are extracted from a feature dataset using trained TextCNN models. TextCNN layers are used to learn distinctive malware representations to enhance the efficiency of the proposed HSAS-MD analyzer. Therefore, CNN’s architecture is designed to extract the features from the dataset by using a five-layered CNN model [56], as shown in Figure 9. The five layers are:

The input layer (C1) is the input feature in the vector-based 7 × 5 matrix. This layer begins with triggers, conditions, and actions, which are then converted into a sentencing matrix; the rows are word-vector representations of each. The sentence length is s and the dimension of the word vector by d; the dimension of the sentence matrix is s × d^2^.

The first hidden layer (C2) is the layer that filters features into six filters. There are three filter region sizes: 2, 3, and 4, each with two filters. The weight matrix w makes the filter with region size z. Then, z · d parameters were obtained. The sentence matrix by k ∈ R^s×d^, k [m: n] represents the sub-matrix of k from row m to row n. The output sequence X ∈ R^s−z+1^ of the convolution operator is obtained by repeatedly applying the filter on sub-matrices of k.

X_i_ = w · k [m: m + z − 1] (1)

where m = 1. s − z + 1, then adds a bias term b ∈ R and an activation function f to each X_i_, inducing the c ∈ R ^s−z+1^ for this filter:

c_i_ = f (X_i_ + b) (2)

The second hidden layer (C3) aims to resample and transform the second layer result. Thus, for each feature map in C2, six filters of 4 × 1 vectors are obtained. This feature map was obtained after applying many filters for the same region size z.

In the third hidden layer (C4), the six filters in c3 are merged, and three filters of 2 × 1 are obtained. Each feature map is applied to 1-max pooling, which means that the most significant number from each feature map is collected. Thus, a simple feature vector is formed from all six maps, and these six features are combined to build a feature vector for the final layer.

The output layer (C5): this is a final layer of CNN, which receives a C4 feature vector to classify the sentences. It will classify them into two classes.

### 4.5. Filtration Phase in the Proposed HSAS-MD

This critical phase is used to improve CNN’s training and enhance the accurate decision for the output. “Binary cross-entropy” function used as the output of CNN consists of two classes (the class with the number 1 indicates the malware class, and the class with the number 0 shows the benign class). The following Equation (1) is used in binary cross-entropy. B is the predicted probability, and A is the binary indicator (o or 1). The output of this phase is the final decision if there is malware or not.

BCE = −(A log (B) + (1 − A) log(1 − B))(3)

### 4.6. Model-Checking Technique in the Proposed HSAS-MD

The model-checking technique (MCT) is the core analysis process in the proposed HSAS-MD analyzer for verifying the behavior of IoT apps. MCT has systematically verified that all states of the given system meet the given properties to verify the app’s behavior. The proposed HSAS-MD verifies the rule model in the static analysis phase as the same analyzers [6,45,46] and verifies CFG in the dynamic analysis phase. For the static analysis phase, which depends on the rule model, MCT depends on rewriting logic (the logic that happens due to the changes of the state and concurrent computations). These computations are formulated from logic (L) to rule model (R), meaning that every state that matches the L will be converted to R [57,58]. In [59], the security rules to LTL were formulated, which were verified using MCT. For the dynamic phase, MCT verifies CFG dynamically using DynAUoy. It enhances the e Alloy specification system, allowing models’ dynamic properties to be specified and analyzed [51].

## 5. Implementation, Evaluation, and Discussion of HSAS-MD

This section presents the implementation, evaluation, and discussion of the proposed HSAS-MD analyzer using different evaluation metrics. The central core of evaluating the proposed HSAS-MD is the ability to (i) extract static and dynamic features from the source code of IoT applications; (ii) detect malware from features’ datasets; (iii) detect APP behavior using MCT.

### 5.1. Description of Tools Used

Experiments of program analysis (IoT APPs) were performed with Eclipse IDE and IntelliJ IDEA 2021.1.1 ×64 on a 2.6 GHz Intel i5-4300M processor and 8 GB RAM laptop, using Oracle’s Java Runtime version 1.8 (64 bit) in its default settings. The Alloy tool used for the model-checking test applied the Graphviz tool for state-model representation. The output of the hybrid analysis will be the input of the CNN model. This CNN model used PyTorch, based on torch library runs with Google Colaboratory.

### 5.2. Implementation of the Proposed HSAS-MD

The proposed HSAS-MD depends on reusing a hybrid analysis analyzer that depends on the model-checker tool, then filtering its output features using the CNN model. This method is the fastest, most accurate, and most applicable to be used. The proposed HSAS-MD implementation reused IoTCOM [6] and then filtered its features using the CNN model. There are two stages to verify the proposed HAS-MD analyzer before using the model-checker tool.

#### 5.2.1. Extracting Rule Model from Static Analysis

This stage depends on reusing the analyzer “IoTCOM” [6]. It depends on using the potential of our formal abstractions by creating a modular model extractor. This depends on Alloy, which is a model-checker tool for representing the standard specifications of IoT apps, such as reflecting the behavior of IoT apps to safely detect security risks and respect cyber and physical channels by using IoT apps’ PA code. It consists of two phases: a behavioral rule extractor and a formal analyzer. The output of the static phase “rule extractor,” is reused in HSAS-MD in the CNN model, and the dynamic analysis output is verified directly to the final phase of MCT.

#### 5.2.2. Testing the Deep-Learning “CNN Model”

The CNN model’s task is to filter fake actions (malware) in the proposed HSAS-MD. It takes the static analysis output: triggers, conditions, and actions to be trained and tested. As shown in Table 3, each application has its own rules. The CNN model deals with triggers, conditions, and actions as sentences. These sentences are represented in a word-embedding vector [56]. The testing phase focuses on the action vector, if the trigger vector and condition vector match the same previously restored trigger and condition vector to the same rule in the considered application. The action must match the new action. If the two actions are matched, it is a benign action, and it will be moved to the next phase (MCT). Otherwise, it will be malware.

**Figure 10:**
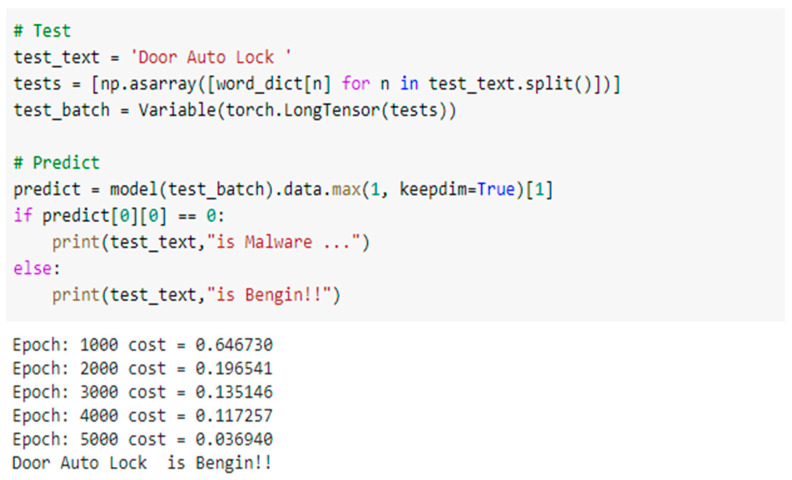
The test of the CNN phase at the HSAS-MD analyzer.

**Figure 11:**
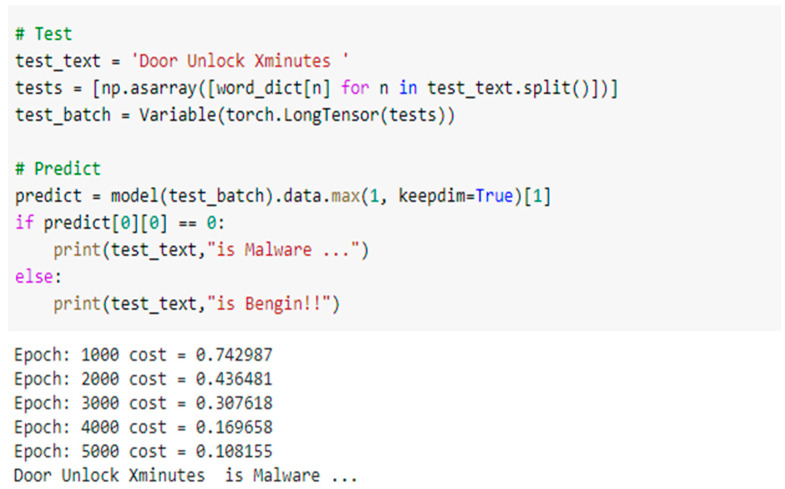
The second test of the CNN phase at the HSAS-MD analyzer.

Figure 10 shows the following test result; when the input action from the considered IoT application “Door Auto-Lock” to CNN is matched, the output of CNN will detect that this application “Door Auto-Lock” is benign. However, Figure 11 shows that the test result is malware. When the input action from this application “Door Unlock X minutes” to the CNN does not match the stored action, it will stop this application from continuing and transferring to the next verifying phase, MCT.

### 5.3. Evaluation Metrics

The performance of the proposed analyzer utilizes the confusion matrix to evaluate the output of the HSAS-MD. The confusion matrix can be made using defined indicators, as shown in Table 4, which provide a helpful way to measure the proposed analyzer’s performance against other SAS analyzers. In this paper, the analyzers were evaluated based on commonly used accuracy, precision, recall, and F1 score, as in Equations (4)–(7), respectively.

**Figure 12:**
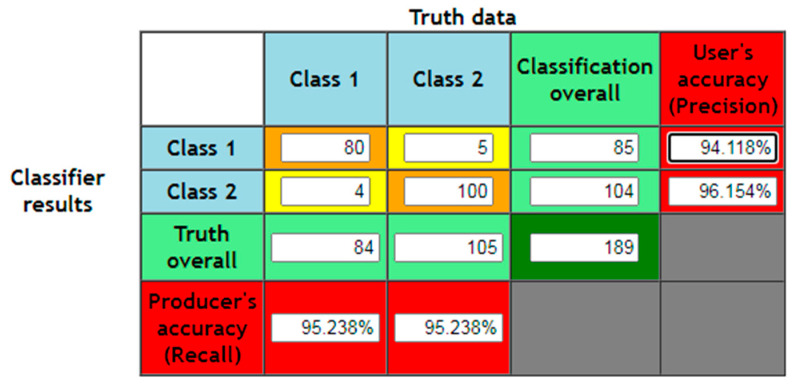
Confusion matrix of the proposed HSAS-MD.

Based on the above indicators, the following performance analysis equations were used to obtain the confusion matrix as shown in Figure 12:

- Accuracy (ACC) is the ratio representing the number of correctly identified analyzers.

ACC = (TP + TN)/(TP + TN + FP + FN)(4)

- Precision (PRC) is the number of accurately predicted true positives.

PRC = TP/(TP + FP)(5)

- Recall (RCL) is the ratio of correctly identified malware relative to the total quantity of malware.

RCL = TP/(TP + FN)(6)

- F-Measure (F-MS) is the ratio that reflects a mixture of accuracy and the recall efficiency of the system.

F-MS = 2 × (Precision × Recall)/(Precision + Recall)(7)

### 5.4. Evaluation of the Proposed Analyzer

To ensure the efficiency of the HSAS-MD analyzer, the evaluation is based on a comparison with competitors’ analyzers. Competitors have been carefully selected to have the same goal to detect malware or to detect abnormal behavior, such as violations due to communication/device failures, physical safety violations, accounts for app interactions, etc., in the source code of the IoT app.

The comparison was established using the same target dataset, “IoTMAL” [60]. This dataset creates erroneous SmartApps with property violations in multi-app or single-app environments. Moreover, more public repository “SmartThings Apps” are used to detect malware. The HSAS-MD used the same test cases of violation detection properties [4,6,10,46] to evaluate the performance, using Table 4.

The following research questions have been created in the evaluation phase of HSAS-MD with related SAS analyzers using Table 5. SAS analyzers were selected based on MCT. Some only use MCT [4,6,10,43,44], while [45,46] use other techniques with MCT.

The proposed HSAS-MD uses two techniques, which are MCT and DL. The following figures [9,10,11,15,16,17,18] are configured to represent the results of the research questions.

**RQ1.**  *What is the accuracy, precision, recall, and F-measure of the proposed HSAS-MD compared to other analyzers?*

**Figure 13:**
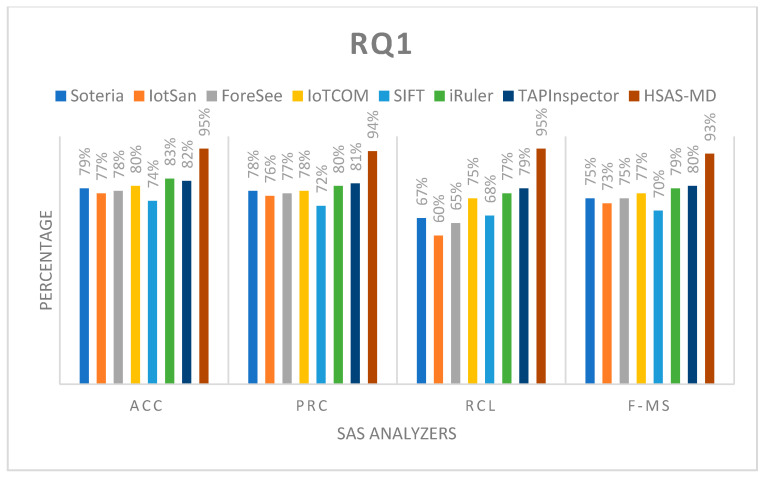
The comparison of the proposed HSAS-MD analyzer with other related analyzers based on ACC, PRC, RCL, and F-MS.

The HSAS-MD gives the best results in accuracy (ACC), precision (PRC), recall (RCL), and F-measure (F-MS), as it depends on using hybrid analysis, not relying only on static or dynamic analysis. This impact plays an essential role in covering all vulnerabilities, as shown in Figure 13. Another impact that makes the proposed HSAS-MD the best is that the state model and rule model are used together in the static phase. It is noted that the analyzers which relied on rule models such as TAPInspector, SIFT, iRuler, and IoTCOM gave the best result than others, such as Soteria, IotSan, and ForeSee, that depend on the state model.

**RQ2.**  *What is the performance, according to the analysis time, to detect abnormal behavior?*

**Figure 14:**
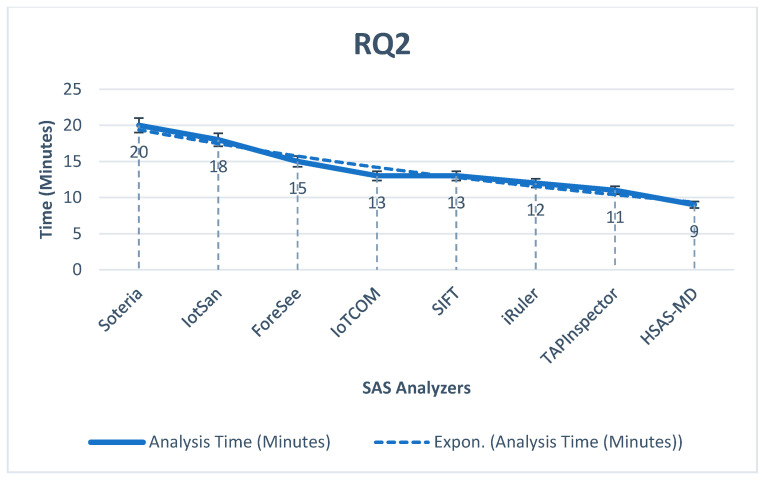
The analysis time of the proposed HSAS-MD analyzer with other related analyzers.

The time of analysis is the essential impact to indicate the performance of analyzers. As shown in Figure 14, The proposed HSAS-MD gives the best performance as it has a fast analysis time for the early detection of malware. The deep-learning CNN model has an essential role in detecting malware early.

**RQ3.**  *What is the performance according to analysis type (static, dynamic and hybrid) in the SAS?*

**Figure 15:**
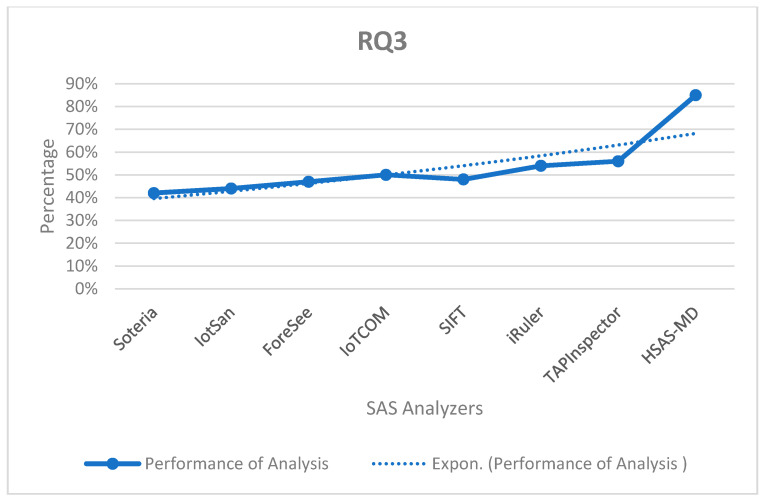
The performance of the proposed HSAS-MD analyzer with other related analyzers based on the analysis type.

The hybrid analysis achieves the best result, as it combines static and dynamic analysis. As shown in Figure 15, the proposed HSAS-MD gives the best results. In contrast, the other analyzers depend on static analysis only.

**RQ4.**  *What is the best the static analysis phase to be used (state model, rule model, and hybrid model that combines state and rule)?*

**Figure 16:**
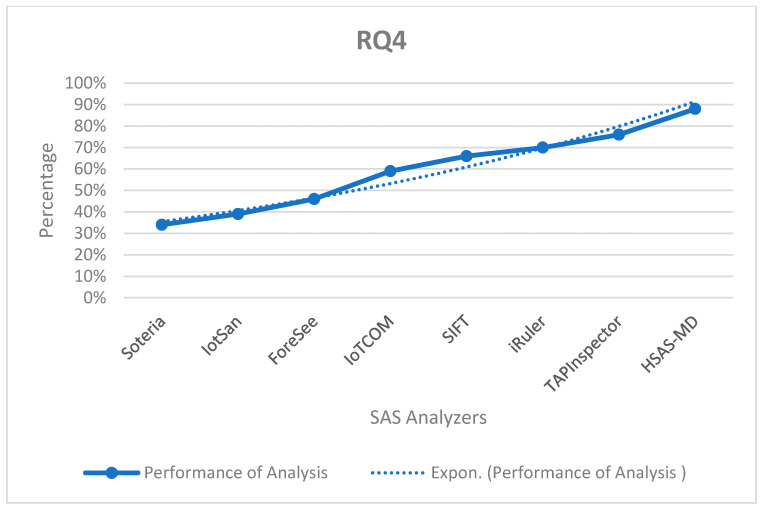
The performance of the proposed HSAS-MD analyzer with other related analyzers based on the static analysis model.

There are three SASs based on the state model: Soteria, IotSan, and ForeSee. Additionally, three SASs are based on the rule model: IoTCOM, SIFT, and iRuler, compared to the proposed HSAS-MD which is based on both the rule and state models. The performance of analyzers increases more using the rule model than the state model. Figure 16 shows that the performance of three SASs based on the rule model is better than the other three SASs based on the state model; the proposed HSAS-MD gives the best performance as it is a hybrid using the two models.

**RQ5.**  *What is the verification time of CNN for detecting conflict rules?*

**Figure 17:**
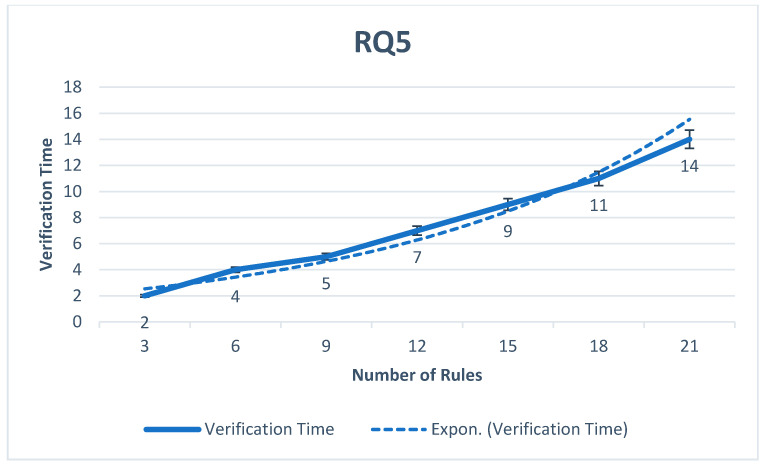
The verification time of the CNN model to verify rules.

**Figure 18:**
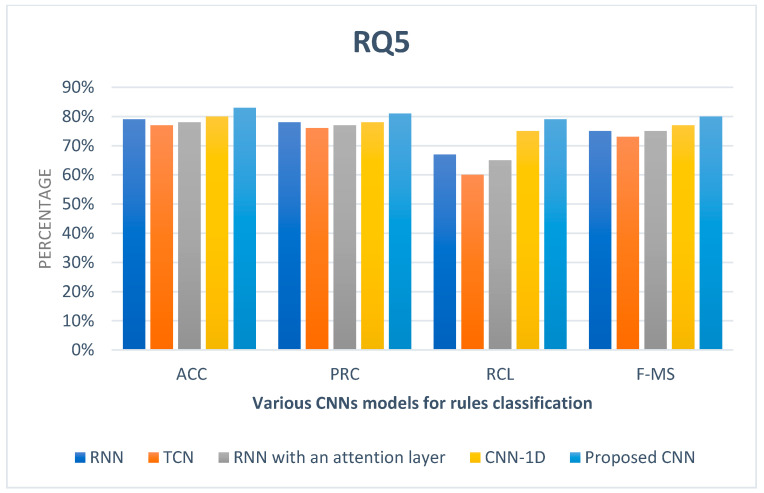
The comparison between various CNN models for rule classification.

It is essential to measure the efficiency of the CNN model. According to rules training and testing in the proposed HSAS-MD, measuring the verification time of CNN is essential. Figure 17 shows that the rate of verification time is suitable to the number of rules used. The verification time increases with the increase in rules at the appropriate time rate. Various CNN models such as RNN [61], TCN [62], RNN with an attention layer [63], and CNN-1D [34] are compared with the proposed CNN to measure the verification time of rules as shown in Figure 18. The proposed HSAS-MD give the best ACC, PRC, RCL, and F-MS to verify and classify rules that may have a fake condition (malware).

## 6. Conclusions, Limitations, and Future Research Trends

The competition between security experts and developers has been a never-ending battle against malware complexities, improving rapidly as innovation advances. It is, therefore, necessary to move towards the development of SASs to ensure the security and safety of IoT applications. This paper presented a hybrid security analysis system (HSAS) based on MCT and DL for IoT applications. The main advantages of the proposed HSAS-MD analyzer are the ability to (i) convert the source code of the target applications to a format of a model checker that can deal with it; (ii) detect any abnormal behavior in the IoT application; (iii) extract the main static features from it to be tested and classified using a deep-learning algorithm; (iv) decide whether the app behavior is safe or not.

It is evident from the experimental results of HSAS-MD that the applied hybrid analysis is better than the static or dynamic analysis, as it provides reliable results and covers all the advantages of static and dynamic analysis. Moreover, the results show that MCT is a more accurate professional analysis technique, particularly with the applied path of sensitivity compared with similar SASs that use various PA techniques. HSAS-MD gives the best results in the detection of physical vulnerabilities and malware.

A conclusion was made about the effectiveness of using hybrid analysis for malware detection, as it covers all issues for detecting malware. It is better than using one type of analysis (static or dynamic analysis). MCT is an efficient technique for detecting malware in applications. In addition, using CNN with MCT give the best results.

There is a limitation in HSAS-MD, which is that malware detection depends on the spatial context, which does not focus on IoT devices’ locations. The physical channels or connections are affected by the location of IoT devices. The second limitation is that HSAS-MD focuses on one type of action conflict, which depends on the value of the actuator. There are two other types of action which rely on time or static attributes, such as sending notifications.

There are more points in future research trends. The first main point addresses the mentioned limitations by focusing on the IoT devices’ locations. Additionally, all types of action conflict will be resolved. The second is enhancing the “program-slicing technique”, which relies on slicing one program into a multi-statement. Using the program-slicing technique with MCT and CNN will provide more accuracy and time. The third point is testing the proposed HSAS-MD with different datasets to increase the training of the detection of new IoT malware. The fourth is that the proposed HSAS-MD will be applied with Android apps for the IoT platform by modifying the specifications that match these Android apps.

## Figures and Tables

**Table 1:** An Overview of Security Analysis Systems (SAS) in IoT applications.

Ref.	SAS. Name	Analysis Type	Analysis Technique	The Main Work	Advantages	Limitations
[17]	Soteria	Static	Model-Checking	Extracts a state model from the code of an IoT application to verify if an application or multi-app system respects security, safety, and functional properties.	SOTERIA analysis on MALIOT indicated it was accurate at recognizing 17 of 20 separate property violations in those 17 apps.	1: The use of call diagnosis by reflection. 2: Dynamic device permissions and app configurations.
[34]	IoTCOM	Static	Model-Checking	Explains how to construct a flexible model extractor that utilizes static analysis algorithms to identify the behavior of IoT apps automatically.	IoTCOM reduces the violation detection time by 92.1%	Depends on all static and dynamic features, which may include unnecessary features.
[5]	IotSan	Static	Model-Checking	Employs the model-checking approach to identify the causes of cyber vulnerabilities and provides actual precedents to clarify such triggers.	Recognizes 147 vulnerabilities. Can identify possible security violations	1: The checker of the Spin model cannot manage a file size more significant than that of the Promela code. 2: The G2J translator does not recognize heterogeneous sets.
[19]	IoTGuard	Dynamic	Code-Instrumentation	Works in three phases: (a) execution of a code instrumentor; (b) storing data of the apps in a dynamic model; (c) detection of IoT security on the dynamic model	IoTGuard introduces 11 single measures, then blocks 16 in 6 (17.1%) Smart-Things, then 5 (16.6%) IFTTT apps.	Enables a user to define policies through IOTGUARD’s GPL.
[30]	PATCH EC-KO	Hybrid	Deep-Learning	Optimizes deep learning and hybrid static-dynamic binary analysis to execute multi-platform binary code similarity analysis to identify vulnerabilities without high-precision source code access.	The PATCHECKO differential engine identifies between still-vulnerable functions and those set with an accuracy of 96%.	The accuracy is not a high ratio.
[21]	IOTFUZZER	Dynamic	Taint-Tracking and Machine-learning	IOTFUZZER, which aims at identifying vulnerabilities to memory corruption in IoT devices without accessing their firmware images.	IOTFUZZER successfully found 15 vulnerabilities of memory corruption (which include eight newly discovered vulnerabilities).	1: Scope of testing. 2: Connection mode 3: Cloud relay 4: Result judgments 5: Result accuracy
[7]	Soteria2	Static	Convolutional Neural network	A random walk-based traversal method for feature extraction employs both density-based and level-based CFG labels to achieve consistent representation.	Soteria achieves a 97.79% accuracy rate for detecting AEs and 99.91% accuracy of malware groups	1: CFG does not necessarily reflect the actual code. 2: Obtaining a CFG representation cannot be performed under obfuscation

**Table 2:** Rule representation model.

No.	Slot Names	Description
1	Rule (Rh)	(trigger) (condition) (action)
2	Trigger (Th)	(capabilities) (attribute) (value)
3	Condition (Ch)	(capabilities) (attribute) (value)
4	Action (Ah)	(capabilities) (attribute) (value)
5	Event	(subject) (attribute)
6	Constraint	logical expression | null

**Table 3:** Rules description of the IoT apps dataset in a smart home.

Application Name	Capabilities	Rule No.	Triggers	Conditions	Actions
AutoLockafter Xminutes	lock1 + state	R0	**capabilities =** app_AutoLockafterXminutes.lock1	**capabilities =** app_AutoLockafterXminutes.lock1	**attribute =** app_AutoLockafterXminutes.state
			**attribute =** cap_lock_attr_lock	**attribute =** cap_lock_attr_lock	**attribute =** cap_runIn_attr_runIn
			no value	**value =** cap_lock_attr_lock_val_locked	**value =** cap_runIn_attr_runIn_val_on
		R1	no triggers	**capabilities =** app_AutoLockafterXminutes.state	**attribute =** app_AutoLockafterXminutes.lock1
				**attribute =** cap_state_attr_runIn	**attribute =** cap_lock_attr_lock
				**value =** cap_state_attr_runIn_val_on	**value =** cap_lock_attr_lock_val_lock
AutoLockDoorsv2	lock1 + state	R0	**capabilities =** app_AutoLockDoorsv2.lock1	**capabilities =** app_AutoLockDoorsv2.lock1	**attribute =** app_AutoLockDoorsv2.state
			**attribute =** cap_lock_attr_lock	**attribute =** cap_lock_attr_lock	**attribute =** cap_runIn_attr_runIn
			no value	**value =** cap_lock_attr_lock_val-cap_lock_attr_lock_val_locked	**value =** cap_runIn_attr_runIn_val_on
		R1	no triggers	**capabilities =** app_AutoLockDoorsv2.state	**attribute =** app_AutoLockDoorsv2.lock1
				**attribute =** cap_runIn_attr_runIn	**attribute =** cap_lock_attr_lock
				**value =** cap_runIn_attr_runIn_val_on	**value =** cap_lock_attr_lock_val_locked
DoorAutoLock	lock1 + state	R0	**capabilities =** app_DoorAutoLock.lock1	**capabilities =** app_DoorAutoLock.lock1	**attribute =** app_DoorAutoLock.state
			**attribute =** cap_lock_attr_lock	**attribute =** cap_lock_attr_lock	**attribute =** cap_runIn_attr_runIn
			no value	**value =** cap_lock_attr_lock_val_unlocked	**value =** cap_runIn_attr_runIn_val_on
		R1	no triggers	**capabilities =** app_DoorAutoLock.state	**attribute =** app_DoorAutoLock.lock1
				**attribute =** cap_runIn_attr_runIn	**attribute =** cap_lock_attr_lock
				**value =** cap_runIn_attr_runIn_val_on	**value =** cap_lock_attr_lock_val_locked
DoorsUnlocked	presence1 + lock1	R0	**capabilities =** app_DoorsUnlocked.presence1	**capabilities =** app_DoorsUnlocked.presence1	**attribute =** app_DoorsUnlocked.lock1
			**attribute =** cap_presenceSensor_attr_presence	**attribute =** cap_presenceSensor_attr_presence	**attribute =** cap_lock_attr_lock
			no value	**value =** cap_presenceSensor_attr_presence_val_null	**value =** cap_lock_attr_lock_val_lock
IfFloodTurnValveOff	alarm + valve	R0	**capabilities =** app_IfFloodTurnValveOff.alarm	**capabilities =** app_IfFloodTurnValveOff.valve	**attribute =** app_IfFloodTurnValveOff.valve
			**attribute =** cap_waterSensor_attr_water	**attribute =** cap_valve_attr_any	**attribute =** cap_valve_attr_valve
			**value =** cap_waterSensor_attr_water_val_wet	**value =** cap_valve_attr_any_val_no_value	**value =** cap_valve_attr_valve_val_closed
ItsTooCold	temperatureSensor1 + switch1	R0	**capabilities =** app_ItsTooCold.temperatureSensor1	**capabilities =** app_ItsTooCold.temperatureSensor1	**attribute =** app_ItsTooCold.switch1
			**attribute =** cap_temperatureMeasurement_attr_temperature	**attribute =** cap_temperatureMeasurement_attr_temperature	**attribute =** cap_switch_attr_switch
			no value	**value =** range_0//cap_temperatureMeasurement_attr_temperature_val_lte_tooCold	**value =** cap_switch_attr_switch_val_on
LightOnCold	temperatureSensor1+ switch1	R0	**capabilities =** app_LightOnCold.temperatureSensor1	**capabilities =** app_LightOnCold.temperatureSensor1	**attribute =** app_LightOnCold.switch1
			**attribute =** cap_temperatureMeasurement_attr_temperature	**attribute =** cap_temperatureMeasurement_attr_temperature	**attribute =** cap_switch_attr_switch
			no value	**Value =** cap_temperatureMeasurement_attr_temperature_val_lte_tooCold	**value =** cap_switch_attr_switch_val_on
Motion TriggersLock	motion1 + lock1	R0	**capabilities =** app_MotionTriggersLock.motion1	**capabilities =** app_MotionTriggersLock.lock1	**attribute =** app_MotionTriggersLock.lock1
			**attribute =** cap_motionSensor_attr_motion	**attribute =** cap_lock_attr_lock	**attribute =** cap_lock_attr_lock
			**value =** cap_motionSensor_attr_motion_val_active	**value =** cap_lock_attr_lock_val_locked	**value =** cap_lock_attr_lock_val_unlocked
DelayedCommandExecution	contact1 + switch1	R0	**capabilities =** app_DelayedCommandExecution.contact1	**capabilities =** app_DelayedCommandExecution.contact1	**attribute =** app_DelayedCommandExecution.switch1
			**attribute =** cap_contactSensor_attr_contact	**attribute =** cap_contactSensor_attr_contact	**attribute =** cap_switch_attr_switch
			no value	**value =** cap_contactSensor_attr_contact_val_open	**value =** cap_switch_attr_switch_val_on
		R1	**capabilities =** app_DelayedCommandExecution.contact1	**capabilities =** app_DelayedCommandExecution.contact1	**attribute =** app_DelayedCommandExecution.switch1
				**attribute =** cap_contactSensor_attr_contact	
			**attribute =** cap_contactSensor_attr_contact	**value =** cap_contactSensor_attr_contact_val -cap_contactSensor_attr_contact_val_open	**attribute =** cap_switch_attr_switch
				**capabilities =** app_DelayedCommandExecution.contact1	
			no value	**attribute =** cap_contactSensor_attr_contact	**value =** cap_switch_attr_switch_val_off
				**value =** cap_contactSensor_attr_contact_val_closed	
UnlockitWhenitOpens	contact1 + Lock1	R0	**capabilities =** app_UnlockitWhenitOpens.contact1	**capabilities =** app_UnlockitWhenitOpens.contact1	**attribute =** app_UnlockitWhenitOpens.lock1
			**attribute =** cap_contactSensor_attr_contact	**attribute =** cap_contactSensor_attr_contact	**attribute =** cap_lock_attr_lock
			no value	**value =** cap_contactSensor_attr_contact_val_open	**value =** cap_lock_attr_lock_val_unlock

**Table 4:** Description of the Confusion Matrix.

Indicator	Description
True positive (TP)	The number of malware samples was detected correctly and labeled as malware.
True negative (TN)	The number of benign samples was correctly detected and labeled as benign.
False positive (FP)	The number of benign samples was wrong and labeled as malicious.
False negative (FN)	The number of malware samples was wrong and labeled as benign.

**Table 5:** A comparison between various SASs based on MCT.

Paper	SAS Analyzers	Analysis Technique	Analysis Type	Static Analysis Model
[10]	Soteria	MCT	Static	State
[4]	IotSan	MCT	Static	State
[43]	ForeSee	MCT	Static	State
[6]	IoTCOM	MCT	Static	Rule
[44]	SIFT	MCT	Static	Rule
[45]	iRuler	MCT & NLP	Static	Rule
[46]	TAPInspector	MCT & Slicing	Static	Rule
This Paper	HSAS-MD	MCT &CNN	Static & Dynamic	State & Rule

## Data Availability

Data supporting reported results can be found by contacting authors.

## Abbreviations

**Symbol**	**Description**
APP	Application
AST	Abstract Syntax Tree
BCE	Binary Cross-Entropy
CFG	Control Flow Graph
CNN	Convolutional Neural Networks
DCR	Device Capability Reference
DGR	Dependency Graph Representation
DL	Deep Learning
ICFG	Inter-procedural control flow graph
IR	Intermediate Representation
MCT	Model-Checking Technique
PA	Program Analysis
SAS	Security Analysis Systems
LTL	Linear Temporal Logic
CTL	Computing Tree Logic
SMT	Satisfiability Modulo Theories
